# Glutamate and tumor-associated epilepsy

**DOI:** 10.18632/oncotarget.350

**Published:** 2011-11-06

**Authors:** Harald Sontheimer

**Affiliations:** University of Alabama Birmingham

For many patients an unexplained seizure is among the earliest symptoms of a growing primary brain tumor. These tumors originate from glial cells or their progenitors in the brain and are often referred to as glioma. In approximately 30% of patients seizures recur spontaneously giving rise to tumor associated epilepsy [1]. While these fits are generally well controlled by antiepileptic medications, in some instances the tumor-associated epilepsy remains refractory to medical treatment [2] and seriously impedes a patient's quality of life. It is commonly assumed that seizures are caused by an imbalance of excitatory glutamatergic and inhibitory GABAergic neurotransmission. Such imbalances may involve structural changes in the brain or changes in the receptor composition of hyperexcitable brain regions. These alterations may be congenital or caused by acute or chronic insults. Tumor-associated epilepsy falls into the latter category, and it had long been assumed that the growing tumor mass exerts a pro-convulsive effect by compressing surrounding tissue. However, a series of recent studies suggest instead that enhanced glutamate excitability may be the major cause for tumor-associated epilepsy.

More specifically, studies conducted *in vitro, in situ*, and *in vivo* suggest that glioma cells assiduously release glutamate at concentrations that can cause neuronal hyperexcitability, edema, and ultimately wide-spread tissue destruction [3, 4]. Glutamate is released via the system x_c_^−^ (SXC) cystine-glutamate exchanger, and neuronal death is mediated by MK801 sensitive neuronal NMDA receptors [3-5]. Therefore the tumor associated destruction is akin to glutamate excitotoxicity, which is well described as the major pathway for neuronal demise in stroke and ALS [6]. The SXC transporter responsible for glutamate release from gliomas is the principle pathway for cystine uptake destined for the synthesis of the cellular antioxidant glutathione. Hence glutamate release from gliomas occurs as an obligatory byproduct of the cellular synthesis of GSH [7].

A recent study that used a murine glioma model suggests that glutamate release from the glioma is directly responsible for epileptic activity and represents an early event in tumor development [8]. Tissue isolated from tumor bearing animals showed increased glutamate release and presented with peritumoral hyperexcitability that propagated into the adjacent brain. Animals treated by i.p. injection with sulfasalazine (SAS), a non-transportable substrate inhibitor of SXC, showed a transient reduction in epileptic activity *in vivo*, and tissue slices from tumor bearing animals showed reduced neuronal hyperexcitabiliy after SAS application [8]. This data makes a strong case for targeting the SXC transporter therapeutically in glioma patients and possibly to consider SAS as an available drug candidate. It is important to emphasize that the consequences of glutamate release are not limited to epileptifom activity but indicate active excitotoxic tissue destruction. Therefore limiting glutamate release holds promise to also slow tumor growth as has been demonstrated in a mouse model of glioma [7].

Since these findings are based on animal models, they require confirmation in humans. A recent study showed that 7/7 glioma patients presented with large elevations in glutamate measured by microdialysis, confirming an important role for glutamate in the etiology and expansion of human gliomas [9]. The fact that SAS is widely used to treat a number of chronic inflammatory bowl disorders and has a well known safety profile makes clinical evaluation in glioma patients readily feasible. Of note, SAS has been proposed as a potential candidate to treat several other cancers including prostate and small lung cell carcinomas [10]. Hence this drug holds promise to be useful as adjuvant treatment for a number of cancers.
